# Analysis of Cells Targeted by *Salmonella* Type III Secretion In Vivo

**DOI:** 10.1371/journal.ppat.0030196

**Published:** 2007-12-21

**Authors:** Kaoru Geddes, Frank Cruz, Fred Heffron

**Affiliations:** 1 Department of Microbiology and Immunology, Oregon Health and Science University, Portland, Oregon, United States of America; 2 Department of Pathology, Oregon Health and Science University, Portland, Oregon, United States of America; Tufts University School of Medicine, United States of America

## Abstract

The type III secretion systems (TTSS) encoded in *Salmonella* pathogenicity island-1 and -2 (SPI-1 and -2) are virulence factors required for specific phases of *Salmonella* infection in animal hosts. However, the host cell types targeted by the TTSS have not been determined. To investigate this, we have constructed translational fusions between the ß-lactamase reporter and a broad array of TTSS effectors secreted via SPI-1, SPI-2, or both. Secretion of the fusion protein to a host cell was determined by cleavage of a specific fluorescent substrate. In cultured cells, secretion of all six effectors could be observed. However, two to four days following i.p. infection of mice, only effectors secreted by SPI-2 were detected in spleen cells. The cells targeted were identified via staining with nine different cell surface markers followed by FACS analysis as well as by conventional cytological methods. The targeted cells include B and T lymphocytes, neutrophils, monocytes, and dendritic cells, but not mature macrophages. To further investigate replication in these various cell types, *Salmonella* derivatives were constructed that express a red fluorescent protein. Bacteria could be seen in each of the cell types above; however, most viable bacteria were present in neutrophils. We find that *Salmonella* is capable of targeting most phagocytic and non-phagocytic cells in the spleen but has a surprisingly high preference for neutrophils. These findings suggest that *Salmonella* specifically target splenic neutrophils presumably to attenuate their microbicidal functions, thereby promoting intracellular survival and replication in the mouse.

## Author Summary

Bacteria of the *Salmonella* genus are important human pathogens and a leading cause of food-borne illness. *Salmonella* species' ability to cause disease relies on the activities of two sophisticated molecular syringes that allow the bacteria to pump proteins into cells that they infect. The activities of these syringes have been studied extensively in cells grown under laboratory conditions and shown to be essential for the infectious process in animal models. However, the specific cells within infected organs that are targeted by these syringes have not been identified. In this work we describe the specific spleen cells targeted by *Salmonella* in the mouse. We find that *Salmonella* is capable of targeting most cell types using their molecular syringes. Quite surprisingly, we find that *Salmonella* mostly targets neutrophils, a cell type not thought to be associated with live *Salmonella* in host tissues. These findings challenge our current views of *Salmonella* infection and may lead to new insight for treating the disease.

## Introduction

The innate and adaptive immune systems of the host present a formidable barrier to infection. To overcome the multi-faceted defenses, microbial pathogens have evolved equally complex mechanisms that are only partially understood. One of these mechanisms is the type III secretion system (TTSS) found in many Gram-negative bacterial pathogens. These are sophisticated secretion devices that inject specific proteins (called effectors) directly into the host cell cytoplasm. Various cell culture models are used to study effectors but the cell types targeted by the *Salmonella* TTSS during the course of infection have not been studied.

Salmonella enterica serovar Typhimurium (referred to as *S. typhimurium* hereafter) has two TTSSs that are expressed under different conditions and required for distinct aspects of infection [1–3]. Effectors secreted by the *Salmonella* pathogenicity island-1 TTSS (SPI-1 TTSS) are associated with the invasion of intestinal epithelial cells and enhanced intestinal inflammation in infected hosts [4–6]. The *Salmonella* pathogenicity island-2 TTSS (SPI-2 TTSS) is required for intracellular survival during the systemic phase of infection [7–11], but it also enhances inflammation during the enteric phase [12,13]. In previous work, effectors could be placed into three categories; those secreted via SPI-1 TTSS only, those secreted by SPI-2 TTSS only, or those secreted by both [14,15]. Additional roles for SPI-1 and SPI-2 are still being found. For example, Lawley et al. found that components of the SPI-1 TTSS are required for persistence in a chronic infection model in 129X1/SvJ mice [16].

Whether *Salmonella* persists or kills its host is determined by several factors such as the route of administration, the strain of *Salmonella*, and the strain of mouse infected. Acute infection is usually studied in Balb/c or C57BL/6 mice because they are as sensitive to S. typhimurium infection as humans are to S. enterica serovar Typhi. In acute mouse infection *Salmonella* moves rapidly to the two filtering organs, the spleen and liver, and within those organs is found in macrophages, neutrophils, and dendritic cells [17–22]. Macrophages are considered the primary reservoir of *Salmonella* because survival within macrophages is an essential virulence mechanism [23]. However, the specific cell types targeted by SPI-1 TTSS and SPI-2 TTSS in vivo have not been identified.

In this study, mice were infected i.p. with strains of S. typhimurium expressing different effector-ß-lactamase (Bla) fusions. This reporter system allows detection of secreted effectors by detecting cleavage of coumarin cephalosporin fluorescein (CCF2-AM) [24,25]. This dye is cell permeant but is rapidly cleaved by endogenous cytoplasmic esterases resulting in a charge moiety that is concentrated within the cell. When cleaved by ß-lactamase the dye no-longer displays fluorescence resonance energy transfer (FRET) altering the emission spectrum from green to blue. Three-laser FACS analysis allowed us to simultaneously identify multiple cell surface markers and CCF2-AM fluorescence thus identifying targeted cells more accurately than was possible in a previous study with *Yersinia* [26]. Our results show that only the SPI-2 TTSS is active in the mouse spleen and that many cell types are infected with viable bacteria.

## Results

### Effector-Bla Fusions Are Secreted into Cultured Cells

Six effector proteins were selected because they display different secretion patterns in cultured cell lines and are directly involved in virulence [4,15,16,27–30]. These secreted proteins were classified into three groups based on their secretion patterns in cultured cells [15]: SPI-1 only, SPI-2 only, and SPI-1/2. The first group, referred to as S1, includes two proteins, both encoded within *Salmonella* pathogenicity island 1: SipA and SptP (although SptP is secreted at low levels through SPI-2 TTSS). The second group, S2, consists of SteC and SseJ. The last group, S1/2, includes SteA and SlrP secreted via both SPI-1 and SPI-2 TTSS.

**Figure 1:**
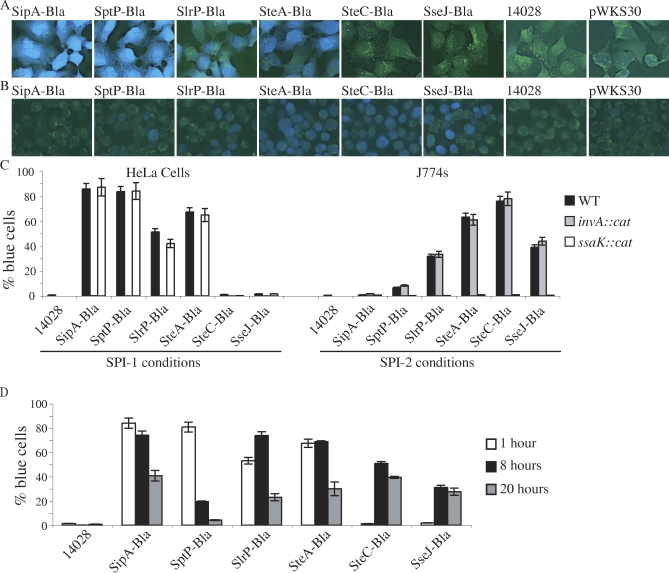
Visualization of Secretion in HeLa Cells (A) and J774s (B) by Microscopy (60× Magnification) S. typhimurium 14028 strains expressing Bla fusions to SipA, SptP, SlrP, SteA, SteC, and SseJ; WT 14028, or 14028 harboring pWKS30 were used to infect HeLa cells for 2 hours using SPI-1-inducing conditions and J774s for 10 hours using SPI-2- inducing conditions. Following the infections, cells were loaded with CCF2-AM and visualized for green and blue fluorescence by microscopy. Green fluorescence indicates CCF2-AM was loaded and the presence of blue cells is evidence of secretion. SPI-1 TTSS- and SPI-2 TTSS-dependent secretion detected by FACS analysis (C). HeLa cells and J774s were infected as described above with the six effector-Bla fusions that were expressed in WT 14028, *invA*::*cat* and *ssaK*::*cat* backgrounds, mutations in structural components of SPI-1 and SPI-2 TTSS, respectively. FACS analysis was performed on CCF2-AM loaded cells to determine the percentage of blue cells (positive for secretion). Background percentage of blue cells was set using WT 14028–infected cells. At least 10,000 cells were analyzed for each sample. Each bar represents the mean percentage of blue cells from triplicate samples and the error bars are ± one standard error of the mean. FACS analysis was also performed on HeLa cells infected with each Bla fusion strain for 1, 8, or 20 hours using SPI-1 inducing conditions (D).

We generated chromosomally encoded effector-Bla fusions in S. typhimurium strain 14028 to the six TTSS effectors listed above and tested them to verify the appropriate secretion patterns in cultured cells. Translational fusions to each of these effectors were expressed from their natural location on the chromosome in order to maintain their native expression patterns. In addition, each fusion was tested to ensure that the derivative retained virulence. For initial experiments, we used cultured cells and infection conditions under which only SPI-1 or SPI-2 TTSS was expressed. The six Bla fusion strains were tested for secretion following infection of HeLa cells for 2 hours and the J774 macrophage-like cell line for 10 hours using either SPI-1 or SPI-2 TTSS inducing conditions, respectively. We found that the expression of the Bla fusions had no effect on the course of infection in these cells lines and that intracellular bacteria were present at all time points investigated. Following the infection, the cells were loaded with CCF2-AM (Invitrogen). Under SPI-1 TTSS inducing conditions, microscopic analysis revealed blue cells among the HeLa cells infected with the S1 and S1/2 fusion strains indicating CCF2-AM cleavage by Bla (Figure 1A). No blue cells were observed in HeLa cells infected with the S2 fusions or in control infections with 14028 or 14028 harboring pWKS30, a plasmid expressing wild-type ß-lactamase protein that is not secreted via TTSS [31]. When SPI-1-dependent infection of HeLa cells was extended to 8 and 20 hours, secretion of S2 effectors was detected (Figure 1D), indicating that the SPI-2 TTSS is active within epithelial cells at these times. Using SPI-2 inducing conditions, blue cells could be seen in J774s infected with the S1/2 and S2 Bla fusions (Figure 1B). A low level of secretion was observed in J774s infected with the SptP-Bla strain. No blue cells were observed in J774s infected with SipA-Bla or control infections. No secretion was observed from strains in which the secretion apparatus had been inactivated (Figure 1C). These results confirmed that secretion by the six Bla fusion strains could be detected in cultured cells under the appropriate infection conditions.

### Detection of Secretion Was Optimal 2 Days following Infection

To test secretion in an in vivo model, C57BL/6 mice were inoculated via i.p. infection with S. typhimurium strains expressing the effector-Bla fusions. Spleens from infected mice were then removed at 24-hour intervals, homogenized, and loaded with CCF2-AM. FACS analysis was performed to detect CCF2-AM cleavage. We were only able to detect secretion in mice when the bacterial load in the spleen was greater than ∼3 × 10^7^ and therefore could not detect secretion within the first 24 hours of infection. If the bacterial load exceeded 1 × 10^8^, the mice became septic resulting in overt splenic cell death making the analysis questionable. Despite this narrow window we could reproducibly recover between 3 × 10^7^ and 1 × 10^8^ bacteria from the spleen 2 days following infection (Figure S1A) and therefore performed all studies using these conditions. By counting viable bacteria recovered from the spleen of infected mice, we confirmed that none of the effector-Bla fusion strains exhibited any defect in their ability to colonize and replicate within the spleen. It is unclear why no secretion could be detected at 24 hours post-infection.

### Red Fluorescent Bacteria Detected within Secretion-Positive “Blue” Cells

**Figure 2:**
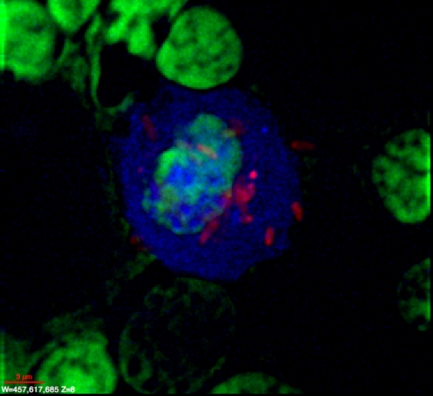
Microscopic Analysis of a Blue Spleen Cell Containing Intracellular *Salmonella* Mice were infected i.p. for 48 hours with 14028 expressing a chromosomal SteA-Bla construct and harboring pWKS30-Tomato, a red fluorescent protein expression vector (shown in red). Spleen cell suspensions were prepared then loaded with CCF2-AM (blue) to detect secretion and a DNA stain, DRAQ5 (green), to visualize nuclei. The image was taken at 60× magnification.

To determine if cells staining blue with CCF2 also contain live *Salmonella* we expressed Tomato red fluorescent protein in each Bla-fusion derivative [32]. Tomato is a red fluorescent protein that has an emission spectrum that does not overlap the other fluorescent dyes used in this work. Splenic cells were scanned by eye for those that fluoresce blue and then examined for the presence of red bacteria (Figure 2). Only cells expressing Bla-fusions to SteA, SlrP, SteC, and SseJ gave positive results. Furthermore, every blue cell identified also contained red-fluorescent bacteria (>100 cells). 3-D reconstruction confirmed that the bacteria associated with these cells were intracellular (Video S1).

### Secretion of S2 Effector-Bla Fusions Was Detected in Splenocytes by FACS

**Figure 3:**
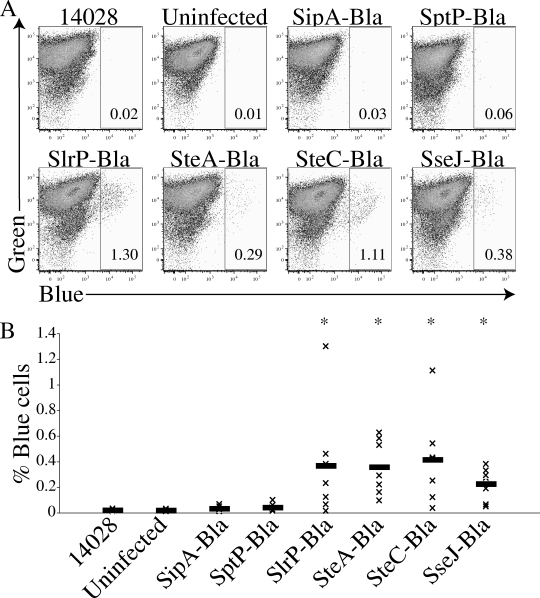
Detection of Secretion in C57BL/6 Spleen Cells FACS data with levels of green and blue CCF2-AM fluorescence in spleen cells from mice infected with each fusion strain (A). Green fluorescence indicates CCF2-AM is present within the cells. The percentage of blue cells, positive for secretion, is shown in the lower right corner of each dot plot. The graph below shows the percentage of total spleen cells emitting blue fluorescence as detected by FACS analysis (B). Each X represents the value for one mouse infected with the indicated strain and horizontal bars represent the average value of seven mice. The * denotes samples for which the Student's *t*-test returned a value where *p* < 0.05 when compared to 14028-infected mice.

At 48, 72, and 96 hours post i.p. infection of *Salmonella*, varying degrees of secretion could be detected by FACS analysis. However, due to the fact that many mice were dead 72 or 96 hours post inoculation, we performed the majority of our analysis after 48 hours. Secretion was detected only in mice infected with strains harboring S1/2 and S2 effector fusions (SteA, SlrP, SteC and SseJ; Figure 3A and 3B). No secretion was detected in mice infected with SipA and SptP effector (S1) fusions. Therefore, the in vivo secretion we observed is likely due to the expression of the SPI-2 TTSS. This finding corresponds well with the virulence data, indicating that SPI-2 but not SPI-1 TTSS is required for spleen colonization and morbidity in this acute mouse infection model (Figure S1B and S1C, Protocol S1). In this same FACS analysis it was not possible to detect internalized bacteria expressing Tomato because the appropriate excitation wavelength was not available. However, bacteria could be detected microscopically following cell sorting of blue fluorescent cells (see below).

### Identification of Spleen Cells Containing ß-Lactamase Reporter Fusions

To identify the cell types that were targeted by S. typhimurium, spleen cells were loaded with CCF2-AM, then stained with several fluorescently conjugated antibodies and analyzed by FACS. Beta-lactamase fusions to SipA or SptP were not analyzed because secretion of these effectors could not be detected in splenic cells (Figure 3). Using FACS analysis, we were able to simultaneously detect four cell-surface markers, as well as blue and green CCF2-AM fluorescence. Each sample was split into two groups and stained with a total of eight fluorescent antibodies. One group was stained with antibodies that recognize cell surface markers specific for T cells and B cells (CD4, CD8, CD3, and CD19) and the second group was stained with antibodies to distinguish macrophages, neutrophils, monocytes and dendritic cells (CD11b, CD11c, GR-1, and F4/80). Parallel samples were stained with propidium iodide to stain dead cells and determine their light scattering properties enabling exclusion of > 90% of dead cells from our analysis. Dead cells do not load with CCF2-AM because retention of this dye requires active cell processes ([25]; Figure S2). Therefore the contribution of autofluorescence from dead cells was minimized.

Table 1 summarizes the percentage of each spleen cell type that was observed in infected and uninfected mice as well as the marker designations used to define each cell type. The percentage of lymphocytes, mature macrophages, monocytes and neutrophils in infected mice, as determined by FACS, corresponded well with the percentages observed by analyzing Wright-Giemsa stained slides. Monocytes found in tissues are considered macrophages. However, we distinguish mature macrophages from monocytes by the presence of tingible bodies on Wright-Giemsa stains as well as by the presence of cell surface markers. Monocytes express intermediate to high levels of both GR-1 and CD11b on their surface whereas mature macrophages do not (our unpublished observation; [33–35]). Table 2 shows the percentage of blue spleen cells represented by specific cell types from mice infected with effector-Bla fusion expressing *Salmonella* strains. There was no statistically significant difference in the cell types targeted by each effector. Secretion could be detected in all cell populations analyzed including CD4 T cells, CD8 T cells, B cells, dendritic cells, monocytes and neutrophils but not mature macrophages. Most of the secretion (∼73% of blue cells) was found in GR-1+/CD11b+ monocytes and neutrophils. Thus, there were several surprising results that warranted additional investigation.

**Table 1:**
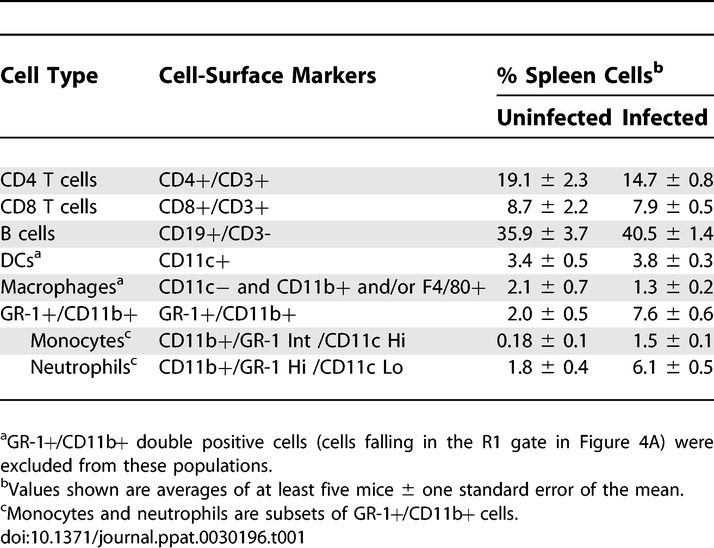
FACS Analysis of Spleen Cells from Uninfected and Infected Mice

**Table 2:**
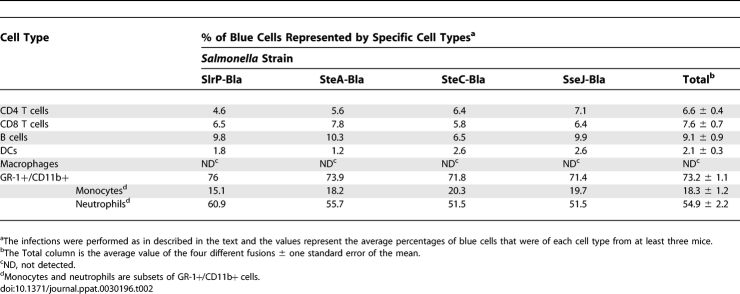
Percentage of Blue Spleen Cells Represented by Specific Cell Types from Mice Infected with Effector-Bla Fusion Expressing *Salmonella* Strains

**Figure 4:**
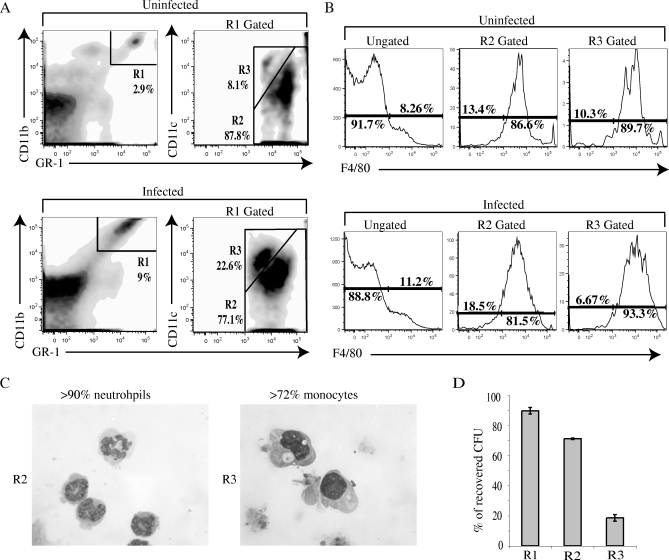
GR-1+/CD11b+ Cells Consist of Neutrophils and Monocytes FACS analysis was performed on spleen cells from uninfected C57BL/6 mice or mice infected with 14028 for 2 days (A). The level of GR-1 and CD11b in total viable spleen cells is shown in the density plots on the left. GR-1+/CD11b+ cells (R1 gate) were analyzed for CD11c and GR-1 expression levels in the density plots to the right. The level of F4/80 expression for ungated cells (all analyzed cells), R2 and R3 gated cells are shown in the histograms (B). GR-1 Hi/CD11c Lo (R2) cells and GR-1 Int/CD11c Hi (R3) cells from infected mice were FACS sorted, then cytospun onto slides, and stained with Wright-Giemsa stain and visualized by microscopy (C). 300 cells from Wright-Giemsa stained slides were analyzed and the percentage of neutrophils and monocytes was determined. The percentage of total intracellular *Salmonella* in GR-1+/CD11b+ cells was estimated (D). GR-1+/CD11b+ cells (R1), GR-1 Hi / CD11c Lo (R2), GR-1 Int / CD11c Hi (R3) populations were FACS sorted, lysed, then plated on LB to determine the number of intracellular CFU. The percentage of total recovered CFU was then calculated for each FACS sorted population (see Materials and Methods). The graph shows the average percentage total recovered CFU present in each FACS sorted population from three mice and the error bars represent one standard error of the mean.

The absence of mature macrophages as targets of type III secretion was puzzling hence we carried out additional controls to verify this conclusion. Spleen cell suspensions were infected with the effector-Bla fusion strains in vitro for 1 hour using SPI-1 TTSS inducing conditions, loaded with CCF2-AM and analyzed by FACS. This control confirmed CCF2-AM cleavage could be detected in all cell types including mature macrophages (Figure S3).

### The GR-1+/CD11b+ Cell Population Consists of Monocytes and Neutrophils

Wright-Giemsa staining of FACS sorted GR-1+/CD11b+ cells from infected mice revealed that these cells consist of neutrophils (∼80%) and monocytes (∼20%) (Figure 4A and 4C). GR-1+/CD11b+ cells were further subdivided into two distinct populations based on the levels of GR-1 and CD11c staining. Wright-Giemsa staining of FACS sorted cells from infected mice demonstrated that GR-1 Int/CD11c Hi cells were mostly monocytes (>72%) and GR-1 Hi/CD11c Lo cells were mostly neutrophils (>90%) (Figure 4C). GR-1 Int/CD11c Hi cells (mostly monocytes) represent ∼18% of the secretion positive cells while the GR-1 Hi/CD11c Lo cells (mostly neutrophils) represent the greatest percentage of the targeted cells (∼55% of blue cells) (Table 2).

These results differ somewhat from previous work based on distinguishing infected cell types with F4/80 [18]. We found that F4/80 is expressed on most GR-1+/CD11b+ cells regardless of CD11c expression during infection (Figure 4B). This indicates that F4/80 cannot distinguish splenic monocytes from neutrophils. However, in uninfected mice, the majority of the spleen cells expressing F4/80 are indeed monocytes and macrophages (Table 1 and Figure 4A). CD115 is also used to distinguish monocytes in uninfected animals. However following infection we detected CD115 on both neutrophils and monocytes (Figure S4). Therefore it appears that infection itself alters the expression pattern of many cell surface proteins including those that are most often used to distinguish monocytes, macrophages and neutrophils.

### GR-1+/CD11b+ Cells Contain Viable Bacteria

The GR-1+/CD11b+ splenic neutrophils and monocytes increased dramatically following infection (Table 1, Figure 4A). These are infiltrating professional phagocytic cells that play a crucial role in innate immunity by directly killing the invading microbe. We wished to determine whether secretion to these cells correlated with the presence of viable intracellular bacteria or whether the cleaved CCF2-AM was by residual ß-lactamase, left over after the *Salmonella* bacteria were killed. GR-1+/CD11b+ cells were FACS sorted, lysed, and plated on LB agar to determine the number of CFU present as compared to the number of CFU from total splenic cells. GR-1+/CD11b+ cells consistently contained most of the total intracellular CFU recovered from the spleen of infected mice (Figure 4D, ∼90% on average). Separation of the cells into neutrophils and monocytes revealed that ∼70% of the intracellular CFUs were associated with neutrophil enriched population while ∼20% were associated with a monocyte-enriched population. Thus, in spite of the fact that neutrophils are very microbicidal, most bacteria were isolated from these cells. We also wished to distinguish between *Salmonella* actively growing within cells versus simply not yet killed. One way to do this is to examine the cells in which cleavage of CCF2 has taken place for the presence of multiple intact bacteria, as described in the next section.

### FACS Sorted Blue Cells Contain Intracellular *Salmonella*

**Figure 5:**
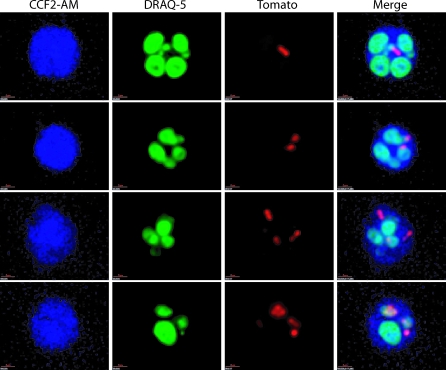
Microscopic Analysis Reveals That FACS Sorted Blue Spleen Cells Contain Intracellular Red-Fluorescent *Salmonella* Mice were infected as described in Figure 2. Spleen cell suspensions were prepared then loaded with CCF2-AM to detect secretion (shown in blue). FACS sorted secretion positive cells (blue cells) were then stained with a DNA stain, DRAQ5 (shown in green), to visualize nuclei. Images were taken at 60× magnification and a single cell is shown in each horizontal row. Red reference bars represent 2 μm.

To determine if cells in which CCF2 had been cleaved contained intracellular bacteria we simultaneously expressed SteA-Bla and Tomato fluorescent protein in the same *Salmonella* derivative. Following infection of C57BL/6 mice, splenic cells were treated with CCF2, and blue fluorescent cells were isolated via FACS sorting. We confirmed by Wright-Giemsa staining that the blue cells were mostly neutrophils and that the remaining cells were a mixture of monocytes and lymphocytes. A fraction of the sorted cells were examined for the presence of intracellular bacteria by fluorescence microscopy. In addition, samples were stained with the nuclear stain, DRAQ5 to aid in distinguishing cell type. Every secretion positive cell that we visualized contained red-fluorescent bacteria that appeared to be intact (Figure 5 and supplemental videos). Most of these cells had nuclei with a multi-lobed appearance suggesting that they were neutrophils (see Videos S2–S5 for visualization of nuclei). Nearly all neutrophils contained multiple bacteria most likely resulting from intracellular replication. The number of infected cells was always <2% of total splenocytes. The low ratio of bacteria to phagocytes indicates a low probability that the same cell could encounter multiple bacteria. Therefore it is unlikely that the presence of multiple bacteria within neutrophils resulted from sequential phagocytic events. Thus, the data suggests that *Salmonella* is replicating within GR-1+/CD11b+ neutrophils.

### *Salmonella* Internalization by B and T Cells Is SPI-1 Independent

**Figure 6:**
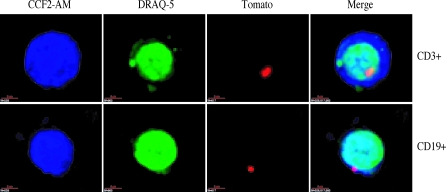
CD3+ and CD19+ Cells Contain *Salmonella* Blue cells that are CD3+ or CD19+ were FACS sorted and visualized for the presence of intracellular red-fluorescent *Salmonella* using fluorescence microscopy. Experiments were performed as described in Figure 5 and red reference bars represent 2 μm.

In tissue culture experiments, SPI-1 TTSS is required for cell invasion in non-professional phagocytic cells. We therefore wished to determine if SPI-1 TTSS were required for internalization by B and T cells and whether SPI-2 TTSS dependent secretion in these cells is associated with intracellular *Salmonella*. To test if uptake by B and T cells occurred via SPI-1 mediated cell invasion the same FACS analysis as above was repeated but in a strain containing a mutation in the SPI-1 structural apparatus (*invA*::*cat*). Mice were infected with this strain or the wild-type control containing both SteA-Bla and Tomato fluorescent protein. Two days after infection cells expressing ß-lactamase and CD19+ (B cells) or CD3+ (T cells) were sorted, stained with DRAQ5, and analyzed by fluorescence microscopy. As expected, sorted cells had nuclear morphology typical of lymphocytes. These cells contained red-fluorescent bacteria for both the SPI-1 mutation and the control (Figure 6). FACS analysis also revealed that secretion into T and B cells occurred at the same frequency in the absence of *invA* (Table S1). These results indicate that *Salmonella* infects non-phagocytic T and B cells in a SPI-1 TTSS independent manner.

## Discussion

This is the first report detailing the cell types targeted by *Salmonella*'s TTSS during a course of infection. Within the spleen a variety of cell types including neutrophils, monocytes, and B and T lymphocytes all showed ß-lactamase cleavage of CCF2-AM demonstrating secretion. Not only were the cells targeted by type III secretion but intact bacteria were also observed in each of these cells. The presence of viable replicating bacteria within monocytes is expected but the presence within neutrophils suggests that *Salmonella* must be defending itself successfully against microbicidal factors present in these cells.

*Salmonella* encodes two different type III secretion systems that play distinct roles by secreting different virulence factors at specific steps during infection. In this work six different secreted effectors were chosen because their secretion patterns were diverse. Two were secreted by SPI-1 TTSS, two secreted by SPI-2 TTSS and two secreted by either apparatus. Although SPI-1 plays a role in a chronic mouse infection [16] it had no effect in our acute infection model. Only SPI-2 TTSS dependent secretion was observed in the mouse spleen after 48 hours of infection. However, we cannot rule out the possibility that SPI-1 TTSS–dependent secretion occurs below our limits of detection, at later time points of infection, in different organs, or into the extracellular space. In a recent report the cell types targeted by *Yersinia* type III secretion were examined with findings that differed from those reported here. These investigators found variations in the cellular targets for secretion of different effectors [26]. In our studies there was no statistical difference in secretion pattern for any of the effectors examined.

### *Salmonella* Targets Non-Phagocytic Lymphocytes

We were surprised to detect secretion of SteA-Bla to splenic B (CD3- and CD19+) and T cells (CD3+ and CD4+ or CD8+). This is not a consequence of auto-fluorescence for several reasons. No blue CCF2-AM fluorescence was observed in B and T cells from mice infected with wild type *Salmonella* (not expressing a Bla fusion). Additionally, dead cells that can exhibit auto-fluorescence were eliminated from analysis using propidium iodide staining to set live cell gates and furthermore live cells were selected by their ability to retain CCF2-AM. Finally, intact *Salmonella* were observed within purified B and T cells. As these are not professional phagocytic cells our first hypothesis was that SPI-1 secreted effectors were stimulating engulfment of the bacteria. However, the percentage of each targeted cell type was unchanged when FACS analysis was carried out with a SPI-1 mutant suggesting that this was not true. Furthermore, using competitive index as a sensitive way of gauging virulence, there was no statistical difference in colonization between the wild type *Salmonella* and a SPI-1 mutant (Figure S1C).

By targeting B and T cells *Salmonella* may influence the development of an adaptive immune response. In fact, infection of B and T cells may account for the course of infection of typhoid fever in man. In typhoid fever, *Salmonella* spreads from primary sites of infection to the bone marrow as well as to other sites [36]. B cells that have been primed by exposure to antigen return to the bone marrow to become blast cells and produce antibody but may be carrying *Salmonella* to the bone marrow as cargo. This may explain why *Salmonella* is a common cause of osteomyelitis.

### *Salmonella* Is Not Found in Mature Splenic Macrophages

*Salmonella* was observed within splenic monocytes but not mature macrophages 48 hours following i.p. infection. Monocytes are immature macrophages that are morphologically distinct from mature macrophages based on Wright-Giemsa staining because they lack ingested material that form tingible bodies typical of mature histiocytes. In addition, monocytes express intermediate to high levels of both GR-1 and CD11b whereas macrophages do not (our unpublished observation; [33–35]). Macrophages were detected among the splenic cells in our studies and could be infected once the cells were isolated (Figure S3). Thus the result is puzzling given the fact that macrophages are professional phagocytes and considered one of the hallmarks of the innate immune response to infection. One possibility is that monocytes are more phagocytic than mature macrophages. In fact a previous study concluded that *Salmonella* targets a specific subset of highly phagocytic macrophages [18]. Another possible explanation is that macrophages are being killed by *Salmonella* shortly after infection. This explanation would also explain the decrease of mature macrophages in the spleen by day 2 of infection (Table 1). Interestingly, Rydstrom and Wick found that splenic monocytes kill *Salmonella* more efficiently than neutrophils [22]. Thus the lack of *Salmonella* in mature macrophages may result from killing by the precursor cell. However this is difficult to reconcile with the fact that *Salmonella* resides in macrophages at later times of infection [21] and that the rate of *Salmonella* killing in vivo is very low [37,38]. Richter-Dalfors et al found that *Salmonella* resides almost exclusively in liver macrophages 5 days post oral inoculation [21] thus it is possible that mature macrophages may simply leave the spleen for other sites.

### Expression of Cell Surface Proteins Changes on Infection

Pathogen associated molecular patterns are detected by the host's innate immune system and result in an immediate response to the infecting organism. The consequence of this is up-regulation of cytokines, chemokines, their receptors, and adhesion molecules. Classically, specific cell surface proteins have been used to distinguish cell types. The drawback of relying on the same cell surface proteins in uninfected and infected hosts is evident in Table 1 and from our other results. This study has the advantage of using two different approaches to distinguish cell types; by fluorescent antibody staining to a variety of cell surface proteins followed by FACS analysis and verification by traditional cytological differentiation after cell sorting. From these two approaches it is clear that cells express a different spectrum of surface proteins after infection than in an uninfected mouse including those that are commonly used to distinguish cell type. As one example, cell markers that were thought to be unique to specific classes of cells such as F4/80 (for macrophages) were found on CD11b+/GR-1+ neutrophils and monocytes.

### *Salmonella* Survives and Replicates within Neutrophils

The current model for *Salmonella* systemic infection is that *Salmonella* reside and replicate within macrophages. However, Dunlap et al. found that most *Salmonella* are within neutrophils 24 hours after i.v. inoculation [20]. Rydstrom and Wick also found *Salmonella* within neutrophils in the Peyer's patches and mesenteric lymph nodes 4 days post oral inoculation [22]. Yet the role of neutrophils in controlling *Salmonella* infection is still controversial. One approach to assess their role in vivo is to deplete neutrophils using specific antibodies and then compare course of infection to an untreated mouse [39–42]. However, depletion studies based on anti-GR-1 deplete not only neutrophils but also monocytes, consequently it is difficult to assess the role of neutrophils [22,35,43]. In vitro, neutrophils kill *Salmonella* only in the first two hours followed by intracellular bacterial replication [44–46] precisely the same pattern as observed for primary macrophages [47–50].

Neutrophils are thought to be a dead end for an intracellular pathogen because they are short-lived and highly microbicidal. They kill bacteria by both oxidative mechanisms (NADPH oxidase and inducible nitric oxide synthetase) and non-oxidative mechanisms (defensins to depolarize the bacterial membrane, neutrophil elastase to destroy components of the type III secretion system, and a protein net to capture and destroy bacteria) [51–54]. However, our data indicates that *Salmonella* not only resides within neutrophils it also replicates within these cells. Our findings challenge the current paradigm that *Salmonella* evades neutrophil killing in vivo by hiding within macrophages. Rather *Salmonella* actively targets neutrophils with type III secretion and subverts them to promote an intracellular niche.

## Materials and Methods

### Bacterial strains and cell culture.

ATCC S. typhimurium 14028 was the parental strain used in all assays. LB media supplemented with antibiotics was used unless otherwise stated. Antibiotics were used at the following concentrations: kanamycin 60 μg/ml, carbenicillin 100 μg/ml. 14028 derivatives and plasmids used in this study are listed in Table S2. J774s and HeLa cells were obtained from ATCC and grown in DMEM supplemented with 10% FBS. HeLa cells and J774s were infected using SPI-1 TTSS and SPI-2 TTSS conditions, respectively, as previously described [15]. For SPI-1 inducing conditions log phase cultures were prepared by sub-culturing overnight cultures 1:33 then growing for three hours with aeration. These cultures were then washed and diluted before infecting HeLa cells at a MOI of 50. For SPI-2 inducing conditions, overnight cultures were washed with PBS then diluted and added to J774s at an MOI of 250. Gentamicin was added to culture medium as previously described [15] to kill extracellular bacteria. To prepare samples for FACS analysis, infected HeLa cells were trypsonized, and infected J774 cells were scraped to prepare cell suspensions, then 10^7^ cells/ml were loaded with .125X solution of CCF2-AM (Invitrogen) for 2 hours at room temperature following the manufacturer's recommendations. To prepare samples of J774 or HeLa cells for microscopy, cells were seeded in Lab-Tek II chamber coverglass slides (Nalge Nunc International), infected, and then 10^7^ adherent cells were loaded with 1 ml of 1X CCF2-AM solution for 2 hours at room temperature.

### Generation of effector-Bla fusions and protein expression analysis.

The mini-Tn5-*bla* transposon was created by replacing the *cyaA'* gene in mini-Tn5-cycler [15] with codons 70–792 of the ß-lactamase gene from pBluescriptSK. This transposon was then used as a template for PCR using primers with extensions specific for the target effector. PCR products were then used to transform 14028 using the λ-red PCR based recombination technique [55] (See Table S3 for a list of primers used). Constructs were confirmed by PCR and by sequencing the PCR products. Each fusion was then transduced into WT 14028, MJW1301 (*ssak::cat*) and MJW1835 (*invA::cat*) backgrounds using P22 phage as previously described [56].

### Preparation of spleen cells.

All mouse studies were approved by the Oregon Health and Science University institutional animal care and use committee (animal protocol #A085). C57BL/6 mice were inoculated via i.p. injection with ∼2 × 10^5^ CFU of the desired S. typhimurium strain. For microscopy, mice were infected with 14028 derivatives carrying plasmid pWKS30-Tomato. Tomato fluorescent protein was obtained from the Tsien laboratory [32]. pWKS30-Tomato was generated by cloning a Shine-Dalgarno sequence and the first half of the tdTomato fluorescent protein into pWKS30. After 2 days of infection, spleens were removed and homogenized using sterile glass slides. The spleen cell suspensions were then digested with collagenase 0.5 mg/ml and DNAse 50 μg/ml in RPMI at 37 °C for 25 minutes. Red blood cells in the spleen cell suspensions were lysed by incubation in a hypotonic red blood cell lysis buffer on ice for 5 minutes. Debris and clumps were removed by straining though a 70-μm cell strainer (BD Falcon). The procedure described above for cultured cells was used to load CCF2-AM. To prepare samples for microscopy, FACS sorted spleen cells were stained with DRAQ5 (Alexis Biochemicals) at a 1:1000 dilution then seeded in Lab-Tek II chamber coverglass slides (Nalge Nunc International).

### FACS analysis and sorting.

To prepare samples for FACS cells loaded with CCF2-AM as described above were first treated with Fc receptor blocking antibodies (see Table S4 for a list of anti-bodies used) for 15 minutes at 4 °C, followed by staining with fluorescently conjugated antibodies at 4 °C for 15 minutes. FACS analysis was performed using an LSRII (Becton Dickinson) FACS analysis machine equipped with 488, 633, and 405-nm lasers. Four cell-surface markers could be simultaneously analyzed along with CCF2-AM fluorescence. Cells were simultaneously stained for four lymphoid specific markers or four myeloid specific markers. The lymphoid specific markers that were analyzed are: CD3, CD4, CD8, and CD19. Myeloid specific markers were: CD11c, CD11b, GR-1, and F4/80 (Table S4). Appropriate isotype control antibodies were used to determine the levels of background staining. Parallel samples were stained with propidium iodide to determine the settings for a live cell gate based on light scatter properties. FlowJo (Tree Star) software was used to analyze the FACS data.

FACS sorting was performed using a FACSVantage SE (Becton Dickinson), with the Digital Vantage option, using the 633, 488 nm, and 405 nm lasers. Spleen cells from infected mice were prepared as described above. Then the cells were stained with antibodies specific myeloid cell surface antigens (CD11c, CD11b, GR-1, and F4/80) or loaded with CCF2-AM and stained with antibodies specific for B and T cell antigens (CD19, CD3). Cells of the desired populations, as described in the results section, were sorted into RPMI media containing gentamicin 100 μg/ml. Reanalysis of sorted cells determined that >99% purity was achieved.

### Cell pathology.

Spleen samples from infected mice were prepared for Wright-Giemsa staining as follows. 1 ml of RPMI containing ∼1 × 10^4^–5 × 10^4^ cells were cytospun onto slides using a Cytospin 2 (Shandon) at 1,500 RPM for 5 minutes. After air-dry fixing, samples were processed for Wright-Giemsa staining [57,58]. Samples were visualized by microscopy and scored for differentials, counting at least 300 cells per sample. Microscopy pictures of Wright-Giemsa stained slides were captured at 100× magnification using a Microphot-FX (Nikon) microscope equipped with a Magnafire (Optronics) camera and using Magnafire (Optronics) software.

### Microscopy on infected cells.

Cells prepared as described above were visualized using 60× or 40× oil-immersion lenses along with emission filter sets for blue (457 nm) and green (528 nm) fluorescence by CCF2-AM, Tomato fluorescent protein (617 nm), and DRAQ5 (685 nm). A UV laser with a DAPI excitation filter was used for CCF2-AM excitation, a 568 nm laser was used for Tomato excitation, and a 647-nm laser was used for DRAQ5 excitation. *z*-sections (0.2 μm) were captured at a resolution of 1,024 by 1,024 pixels. Images were acquired by Aurelie Snyder of the OHSU-MMI Research Core Facility (http://www.ohsu.edu/core/) with an Applied Precision DeltaVision image restoration system. This includes an API chassis with a precision motorized XYZ stage, a Nikon TE200 inverted fluorescence microscope with standard filter sets, halogen illumination with an API light homogenizer, a CH350L camera (500 kHz, 12-bit, 2 Mp, KAF 1400 GL, 1,317 × 1,035, liquid cooled), and DeltaVision software. Deconvolution using the iterative constrained algorithm of Sedat and Agard, and additional image processing were performed using Softworx Explorer Suite (Applied Precision) image processing software.

### Determining percentage of intracellular bacteria in various cell populations.

To determine intracellular CFUs, non-specifically sorted spleen cells (viable cell gate based on light scatter properties) or specifically sorted spleen cell populations were incubated in RPMI containing gentamicin 100 μg/ml ∼2 hours. The cells were then lysed in PBS with 1% Triton X-100 (Sigma) and serial dilutions were plated on LB agar to determine the number of CFU of S. typhimurium present per 10^5^ host cells. To determine the percentage of intracellular bacteria present in different cell populations, the following formula was used: [(CFU/10^5^ specifically sorted spleen cells) × F1] / [(CFU/10^5^ nonspecifically sorted spleen cells) × F2] × 100. Where F1 and F2 are the fractions that a specifically sorted cell population or a non-specifically sorted cell population, respectively, represent out of the total spleen cell population. For example, if the CD11b+/GR-1 Hi/CD11c Lo (neutrophils) cell population represented 6% (F1 = 0.06) of the total spleen cells and contained ∼40 CFU / 10^5^ cells and if in the same mouse, the non-specifically sorted spleen cells contain ∼10 CFU / 10^5^ cells and represent 33.4% (F2 = 0.334) of the total spleen cells. Then using the formula above we would calculate: (40 × 0.06) / (10 × 0.334) × 100 = 72%.

## Supporting Information

Figure S1 Analysis of Mice Infected i.p. with an Inoculum of 2 × 10^5^
The number of bacteria present in the spleen at 24-h intervals following S. typhimurium 14028 infection was determined using at least three mice per time point (A). At 72 hours only nine of 14 mice survived and at 96 h only three out of seven mice survived. The graph shows the log of the number of bacteria present ± one standard error of the mean. Mouse survival assays comparing WT 14028, *invA::cat* and *ssaK::cat* were performed using five mice per group. (B). The percentage of mice surviving at 24-h intervals following inoculation is shown. 4/5 mice infected with *ssaK::cat* survived for 28 days (not shown). Competitive infections were performed mixing WT 14028, *invA::cat* or *ssaK::cat* with MA6054 at a 1:1 ratio and by mixing *ssaK::cat* with *invA::cat* expressed in the same background as MA6054 at a 1:1 ratio (C). 2 days following infection, the number of both strains of bacteria present in the spleen was determined and used to calculate the competitive index (CI) (see Protocol S1). The graph shows the mean CI from five mice ± one standard error of the mean. The * indicates that the Student's *t*-test returned a value of *p* < 0.0001 when compared to the control infection.
(34 KB PDF)

Figure S2 CCF2-AM Is Only Efficiently Loaded into Live Cells Spleen cell suspensions were prepared from infected mice and loaded with CCF2-AM. Dead cells (left panel) and live cells (right panel) were gated based on light scatter properties then analyzed for green CCF2-AM fluorescence. The histograms show the number of cells on the *y*-axis and the relative green fluorescence intensity, indicating CCF2-AM loading, on the *x*-axis.
(17 KB PDF)

Figure S3 CCF2-AM Cleavage Detected in All Spleen Cell Types Spleen cell suspensions were infected with the SteA-Bla strain for 1 hour using SPI-1 inducing conditions. Spleen cells were loaded with CCF2-AM, stained with antibodies, then analyzed by FACS.
(25 KB PDF)

Figure S4 CD115 Expression on GR-1+/CD11b+ Neutrophils and Monocytes FACS analysis was performed on spleen cells from mice infected with 14028 for 2 days. The level of GR-1 and CD11b in total viable spleen cells is shown in the density plots on the left (A). GR-1+/CD11b+ cells (R1 gate) were analyzed for CD11c and GR-1 expression levels in the density plots to the right. The level of CD115 expression for ungated cells (all analyzed cells), R2 (neutrophils), and R3 (monocytes) gated cells are shown in the histograms (B).
(32 KB PDF)

Protocol S1 Supplemental Methods for Mouse Infection Studies (23 KB DOC)

Table S1 Percentage of Blue Spleen Cells Represented by Specific Cell Types from Mice Infected with *Salmonella* Expressing the SteA-Bla Fusion in an *invA::cat* Background (21 KB DOC)

Table S2 Strains and Plasmids Used in This Study (41 KB DOC)

Table S3 Primers Used in This Study (32 KB DOC)

Table S4 Antibodies Used for FACS Analysis (29 KB DOC)

Video S1 3-D Rotation of a Blue Spleen Cell Containing Red Fluorescent *Salmonella*
(475 KB MOV)

Video S2 Example of a Sorted Secretion Positive Cell with a Polymorphonuclear Appearance That Contains Red Fluorescent *Salmonella*
3-D rotation of the cell shown in the top row of Figure 4.
(2.6 MB MOV)

Video S3 Example of a Sorted Secretion Positive Cell with a Polymorphonuclear Appearance That Contains Red Fluorescent *Salmonella*
3-D rotation of the cell shown in the second row (from the top) of Figure 4.
(2.3 MB MOV)

Video S4 Example of a Sorted Secretion Positive Cell with a Polymorphonuclear Appearance That Contains Red Fluorescent *Salmonella*
3-D rotation of the cell shown in the third row (from the top) of Figure 4.
(2.3 MB MOV)

Video S5 Example of a Sorted Secretion Positive Cell with a Polymorphonuclear Appearance That Contains Red Fluorescent *Salmonella*
3-D rotation of the cell shown in the bottom row of Figure 4.
(2.4 MB MOV)

### Accession Numbers

PubMed nucleotide or protein accession numbers are SipA: Nucleotide accession number AF458099, SptP: Nucleotide accession number U63293, SlrP: Nucleotide accession number AF127079, SseJ: Nucleotide accession number AF294582, SteA (STM1583): Protein accession number AAL20501, SteC (STM1698): Protein accession number AAL20615.

## Abbreviations

Bla: ß-lactamase
Group S1: proteins secreted via SPI-1 TTSS: SipA and SptP
Group S1/2: proteins secreted via both SPI-1 and SPI-2 TTSS: SteA and SlrP
Group S2: proteins secreted via SPI-2 TTSS: SteC and SseJ
SPI-1: *Salmonella* pathogenicity island-1
SPI-2: *Salmonella* pathogenicity island-2
*S. typhimurium,*: *Salmonella enterica* serovar Typhimurium
TTSS: type III secretion system
