# Supplementary material for: Analysis of Cells Targeted by Salmonella Type III Secretion In Vivo
Source: PLoS Pathog. 2007 Dec 21;3(12):e196. doi: 10.1371/journal.ppat.0030196 (PMC2151088; doi:10.1371/journal.ppat.0030196)
Supplement: Supplementary file 1 [file ppat.0030196.sg001.pdf]

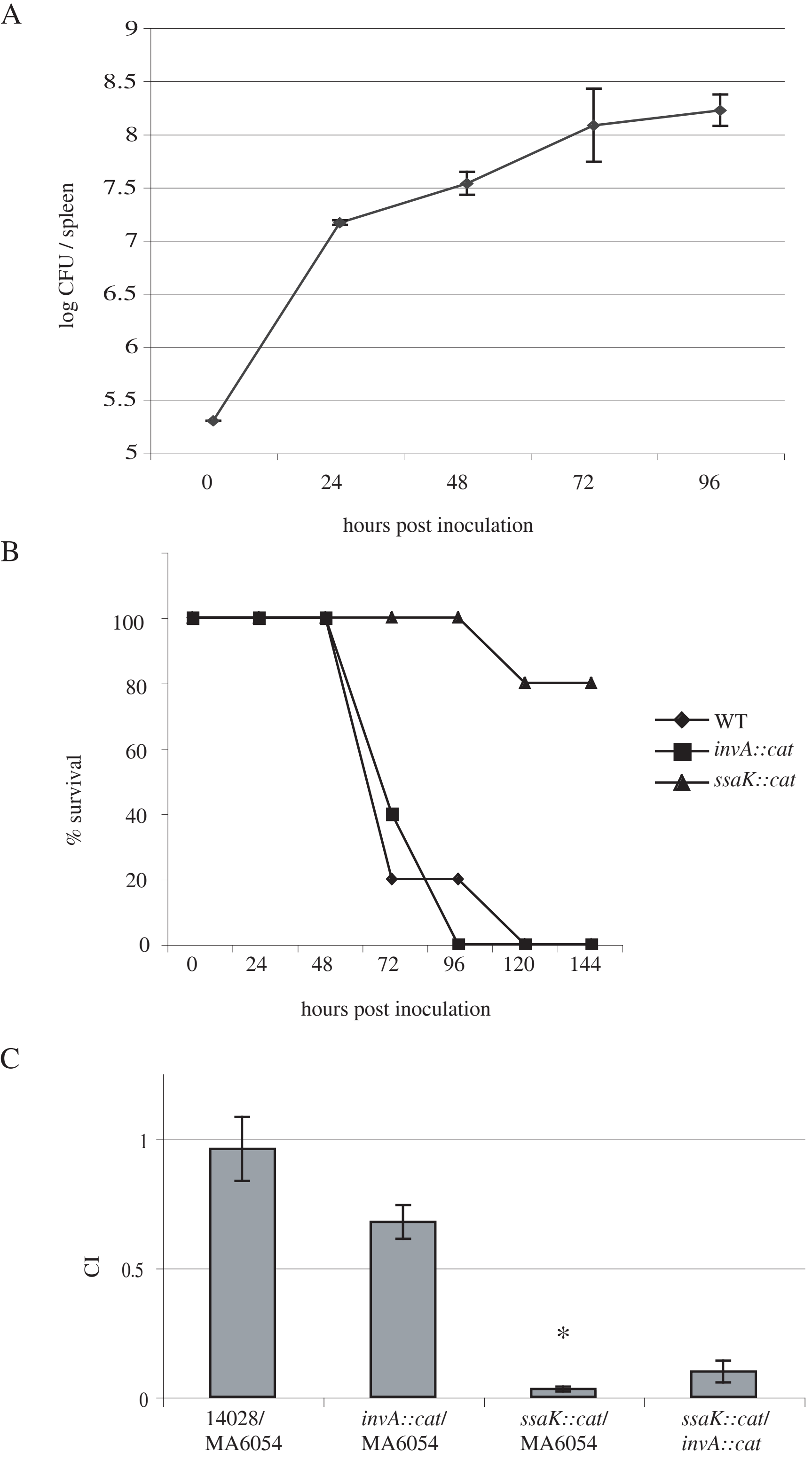

A
log CFU / spleen
9
8.5
8
7.5
7
6.5
6
5.5
5
0
24
48
72
96
hours post inoculation
B
% survival
100
80
60
40
20
0
0
24
48
72
96
120
144
hours post inoculation
WT
invA::cat
ssaK::cat
C
CI
1
0.5
0
*
14028/
MA6054
invA::cat/
MA6054
ssaK::cat/
MA6054
ssaK::cat/
invA::cat
