# Supplementary material for: Analysis of Cells Targeted by Salmonella Type III Secretion In Vivo
Source: PLoS Pathog. 2007 Dec 21;3(12):e196. doi: 10.1371/journal.ppat.0030196 (PMC2151088; doi:10.1371/journal.ppat.0030196)
Supplement: Supplementary file 2 [file ppat.0030196.sg002.pdf]

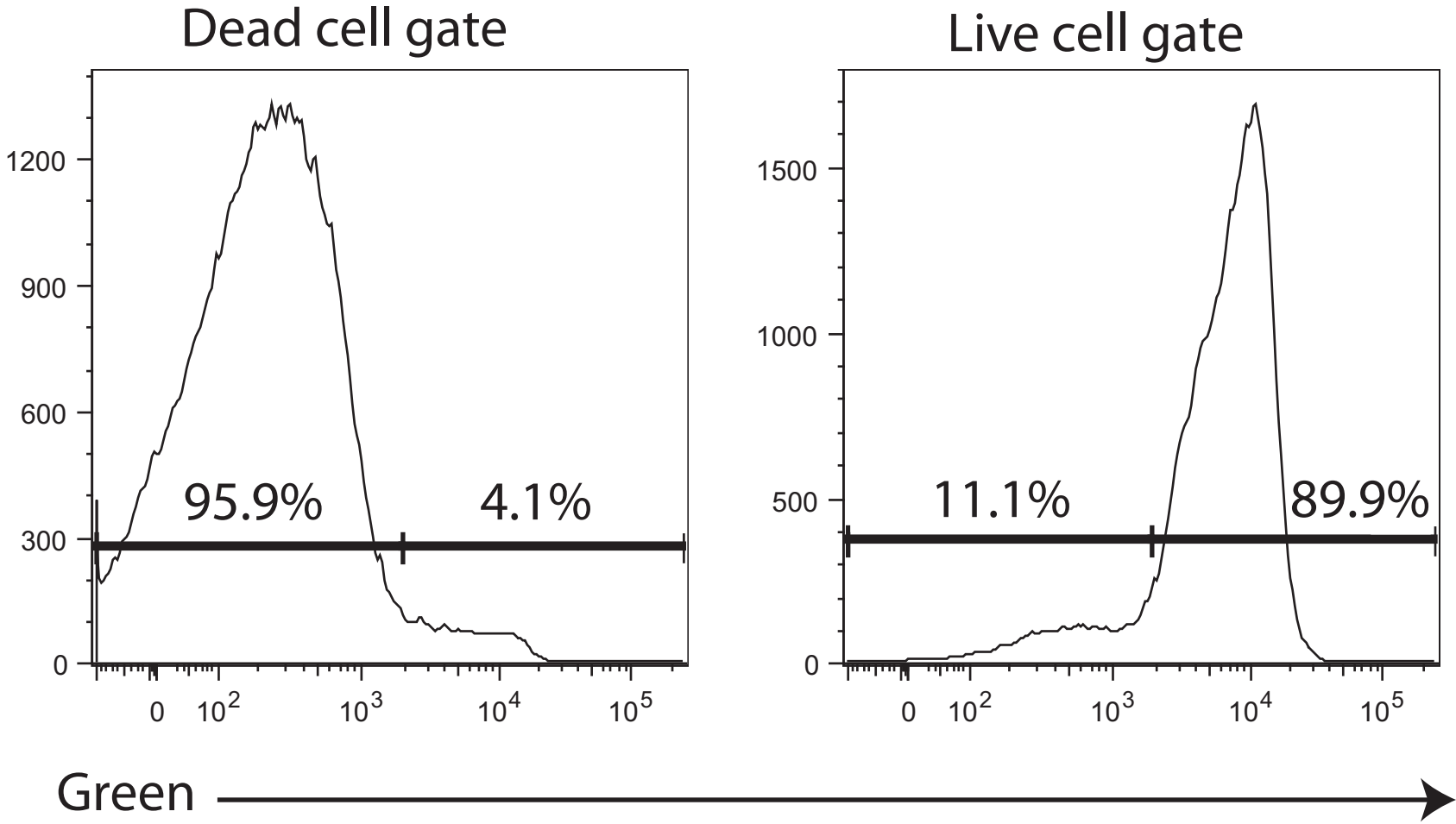

Dead cell gate
Live cell gate
1200
900
600
300
0
95.9%
4.1%
1500
1000
500
0
11.1%
89.9%
0
10^2
10^3
10^4
10^5
0
10^2
10^3
10^4
10^5
Green
