# Supplementary material for: Analysis of Cells Targeted by Salmonella Type III Secretion In Vivo
Source: PLoS Pathog. 2007 Dec 21;3(12):e196. doi: 10.1371/journal.ppat.0030196 (PMC2151088; doi:10.1371/journal.ppat.0030196)
Supplement: Supplementary file 3 [file ppat.0030196.sg003.pdf]

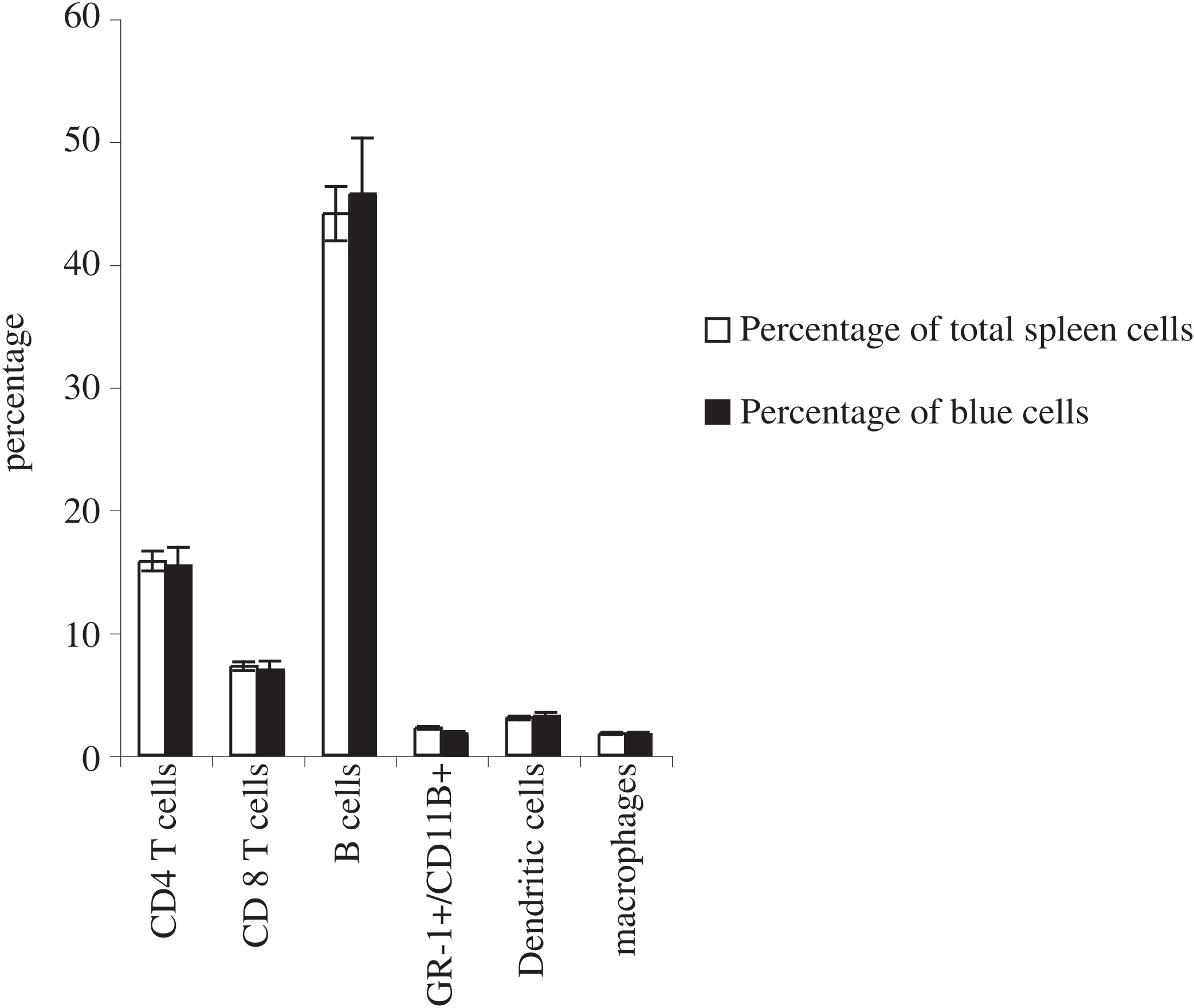

60
50
40
30
20
10
0
percentage
CD4 T cells
CD 8 T cells
B cells
GR-1+/CD11B+
Dendritic cells
macrophages
Percentage of total spleen cells
Percentage of blue cells
