# Supplementary material for: Analysis of Cells Targeted by Salmonella Type III Secretion In Vivo
Source: PLoS Pathog. 2007 Dec 21;3(12):e196. doi: 10.1371/journal.ppat.0030196 (PMC2151088; doi:10.1371/journal.ppat.0030196)
Supplement: Supplementary file 4 [file ppat.0030196.sg004.pdf]

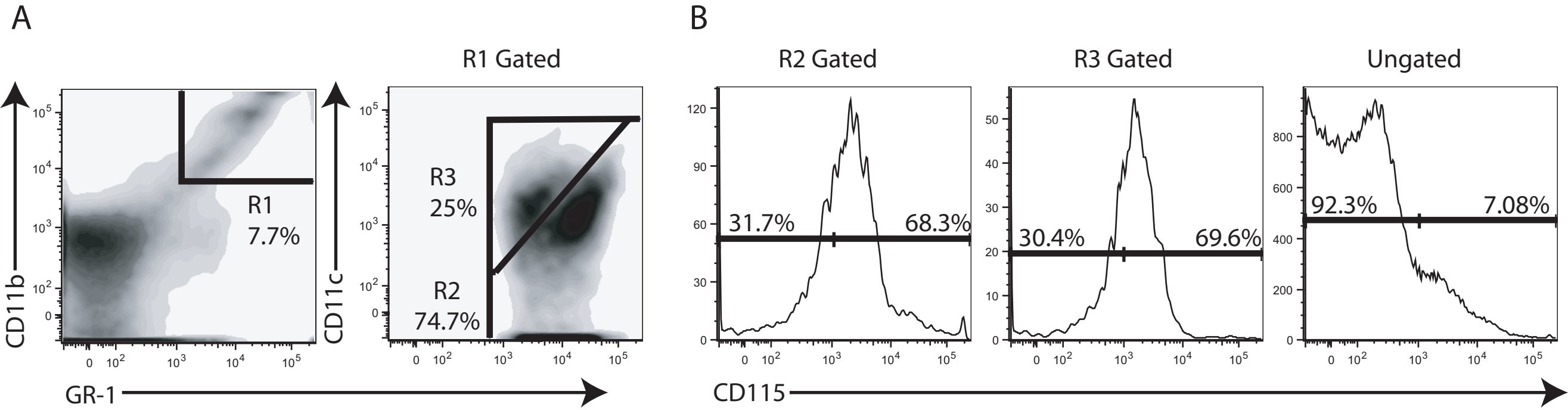
A
B
R1 Gated
R2 Gated
R3 Gated
Ungated
CD11b
CD11c
GR-1
CD115
R1
7.7%
R3
25%
R2
74.7%
31.7%
68.3%
30.4%
69.6%
92.3%
7.08%
