# Supplementary material for: Analysis of Cells Targeted by Salmonella Type III Secretion In Vivo
Source: PLoS Pathog. 2007 Dec 21;3(12):e196. doi: 10.1371/journal.ppat.0030196 (PMC2151088; doi:10.1371/journal.ppat.0030196)
Supplement: Supplementary file 5 [file ppat.0030196.sd001.doc]

**Supplemental methods for mouse infection studies**

All C57BL/6 mice were inoculated via i.p. injection with ~2 X 10^5^ CFU of the desired *S. typhimurium* strain using an inoculum of 100 µl. To determine the number of bacteria present in the spleen, mice were euthanized at the indicated time points and spleens were removed and homogenized. Spleen cells were lysed with PBS containing 1% triton and serial dilutions of this solution were plated on LB agar and colonies were counted the next day. The competitive infections were performed as previously described [15,59]. Each test strain was mixed 1:1 with the reference strain MA6054. 2 days after injection the mice were sacrificed and their spleens harvested and homogenized. Spleen suspensions were diluted and plated on LB plates containing X-gal (40 µg/ml) and arabinose (1mM). The reference strain MA6054 has arabinose inducible β-galactosidase activity and can be easily distinguished from the test strains when plated on LB with X-gal and arabinose. The competitive index (CI) was then calculated using the following equation: (percentage of test strain recovered / percentage of reference strain recovered) / (percentage of test strain inoculated / percentage of reference strain inoculated).
