# Supplementary material for: Analysis of Cells Targeted by Salmonella Type III Secretion In Vivo
Source: PLoS Pathog. 2007 Dec 21;3(12):e196. doi: 10.1371/journal.ppat.0030196 (PMC2151088; doi:10.1371/journal.ppat.0030196)
Supplement: Supplementary file 6 [file ppat.0030196.st001.doc]

Supplemental Table I. Percentage of blue cells represented by different cell types following infection with SteA-BLA expressed in a *invA::cat* background

|  | % of blue cells represented by specific cell types | |
| --- | --- | --- |
| Cell type | CD4 T cells | 5.3 |
|  | CD8 T cells | 8.4 |
|  | B cells | 7.8 |
|  | DC | 1.6 |
|  | Macrophages | ND |
|  | GR-1+ CD11b+ | 71.6 |
|  | Monocytes | 21.1 |
|  | Neutrophils | 50.5 |

ND, Not detected
