# Supplementary material for: Analysis of Cells Targeted by Salmonella Type III Secretion In Vivo
Source: PLoS Pathog. 2007 Dec 21;3(12):e196. doi: 10.1371/journal.ppat.0030196 (PMC2151088; doi:10.1371/journal.ppat.0030196)
Supplement: Supplementary file 7 [file ppat.0030196.st002.doc]

| Supplemental Table II. Strains and plasmids used in this study. | |  |
| --- | --- | --- |
|  |  |  |
| Strain or plasmid | Genotype | Source or reference |
| Strains |  |  |
| 14028s | Wild type *Salmonella enterica serovar* Typhimurium | ATCC |
| JKG100 | Full length chromosomal SseJ::Mini-TN5-BLAM (SseJ-BLAM) in 14028s | This work |
| JKG101 | Full length chromosomal SseJ::Mini-TN5-BLAM (SseJ-BLAM) in MJW1835 | This work |
| JKG102 | Full length chromosomal SseJ::Mini-TN5-BLAM (SseJ-BLAM) in MJW1301 | This work |
| JKG200 | Full length chromosomal SlrP::Mini-Tn5-BLAM (SlrP-BLAM) in 14028s | This work |
| JKG201 | Full length chromosomal SlrP::Mini-Tn5-BLAM (SlrP-BLAM) in MJW1835 | This work |
| JKG202 | Full length chromosomal SlrP::Mini-Tn5-BLAM (SlrP-BLAM) in MJW1301 | This work |
| JKG300 | Full length chromosomal SptP::Mini-Tn5-BLAM (SptP-BLAM) in 14028s | This work |
| JKG301 | Full length chromosomal SptP::Mini-Tn5-BLAM (SptP-BLAM) in MJW1835 | This work |
| JKG302 | Full length chromosomal SptP::Mini-Tn5-BLAM (SptP-BLAM) in MJW1301 | This work |
| JKG400 | Full length chromosomal SipA::Mini-Tn5-BLAM (SipA-BLAM) in 14028s | This work |
| JKG401 | Full length chromosomal SipA::Mini-Tn5-BLAM (SipA-BLAM) in MJW1835 | This work |
| JKG402 | Full length chromosomal SipA::Mini-Tn5-BLAM (SipA-BLAM) in MJW1301 | This work |
| JKG500 | Full length chromosomal SteA::Mini-Tn5-BLAM (SteA-BLAM) in 14028s | This work |
| JKG501 | Full length chromosomal SteA::Mini-Tn5-BLAM (SteA-BLAM) in MJW1835 | This work |
| JKG502 | Full length chromosomal SteA::Mini-Tn5-BLAM (SteA-BLAM) in MJW1301 | This work |
| JKG600 | Full length chromosomal SteC-BLAM (SteC-BLAM) in 14028s | This work |
| JKG601 | Full length chromosomal SteC-BLAM (SteC-BLAM) in MJW1835 | This work |
| JKG602 | Full length chromosomal SteC-BLAM (SteC-BLAM) in MJW1301 | This work |
| JKG1121 | 14028s *ara-907 araD 901*::MudJ *invA*::*cat* | This work |
| MA6054 | 14028s *ara-907 araD 901*::MudJ | [59] |
| MJW1301 | 14028s *ssaK::cat* | [15] |
| MJW1835 | 14028s *invA::cat* | [15] |
| Plasmids |  |  |
| pMini-Tn5-BLAM | pCRScript + mini-Tn5-BLAM | This work |
| pWKS30 | Low copy cloning vector with pSC101 origin and ampicillin resistance gene | [31] |
| pWKS30-Tomato | pWKS30 + Tomato (fluorescent protein) | This work |
|  |  |  |
