# Supplementary material for: Analysis of Cells Targeted by Salmonella Type III Secretion In Vivo
Source: PLoS Pathog. 2007 Dec 21;3(12):e196. doi: 10.1371/journal.ppat.0030196 (PMC2151088; doi:10.1371/journal.ppat.0030196)
Supplement: Supplementary file 8 [file ppat.0030196.st003.doc]

| Supplemental Table III. Primers used in this study. | |  |
| --- | --- | --- |
|  |  |  |
| Primer name | Sequence | Purpose |
| Tn5-*bla* frwd (NdeI) | CATATGGGCACCCAGAAACGCTGGTGAAAGTA | Cloning codons 70-792 of the ß-lactamase gene from pBluescriptSK |
| Tn5-*bla* reverse (BamHI) | GGATCCTTACCAATGCTTAATCAGTGAGGCA | Cloning codons 70-792 of the ß-lactamase gene from pBluescriptSK |
| LacZ-a fusion verification primer | GCTTTTGTTCCCTTTAGTGAGG | Verifying ß-lactamase insertions (in combination with verification primers for specific fusions) |
| Full length SseJ-Bla forward | TGTTAGAAAGTTTTATAGCTCATCATTATTCCACTGAA CTGTCTCTTATACACATCTCA | For amplifying Mini-Tn5-*bla* to make chromosomal full length SseJ-Bla fusion |
| Full length SseJ-Bla reverse | GAGCTGTGTTTTGCTCAAGGCGTACCGCAGCCGATGGAACTCTGTCTCTTATACACATCTGGT | For amplifying Mini-Tn5-*bla* to make chromosomal full length SseJ-Bla fusion |
| SseJ fusion verification primer | CTGTCTCTTATACACATCTGGT | Verifying the full length SseJ-Bla fusion |
| Full length SlrP-Bla forward | AAAAAGAGGTGAGCTCGCTCATGAGCGCCTACTGGCGA CTGTCTCTTATACACATCTCA | For amplifying Mini-Tn5-*bla* to make chromosomal full length SlrP-Bla fusion |
| Full length SlrP-Bla reverse | GGTAAACAGGCTCTCTCCCTCTTCTGATAAACTGCGTTCAGCTGTCTCTTATACACATCTGGT | For amplifying Mini-Tn5-*bla* to make chromosomal full length SlrP-Bla fusion |
| SlrP fusion verification primer | AGAATCAGTTACGAGAGTCGCT | Verifying the full length SlrP-Bla fusion |
| Full length SptP-Bla forward | AAGCAATGCAAGCCCAGTTGCTTATGACGACGGCAAGC CTGTCTCTTATACACATCTCA | For amplifying Mini-Tn5-*bla* to make chromosomal full length SptP-Bla fusion |
| Full length SptP-Bla reverse | AGCTTACTTTCAGATAGTTCTAAAAGTAAGCTATGTTTTTACTGTCTCTTATACACATCTGGT | For amplifying Mini-Tn5-*bla* to make chromosomal full length SptP-Bla fusion |
| SptP fusion verification primer | CTGTCTCTTATACACATCTGGT | Verifying the full length SptP-Bla fusion |
| Full length SipA-Bla forward | GGGTTATTACTACCGTTGATGGCTTGCACATGCAGCGT CTGTCTCTTATACACATCTCA | For amplifying Mini-Tn5-*bla* to make chromosomal full length SipA-Bla fusion |
| Full length SipA-Bla reverse | CTTGCTTCAATATCCATATTCATCGCATCTTTCCCGGTTAACTGTCTCTTATACACATCTGGT | For amplifying Mini-Tn5-*bla* to make chromosomal full length SipA-Bla fusion |
| SipA fusion verification primer | CTGTCTCTTATACACATCTGGT | Verifying the full length SipA-Bla fusion |
| Full length SteA-Bla forward | GCCGACATAAAAGCTCGCTACCATAACTATTTGGACAATTAT CTGTCTCTTATACACATCTCA | For amplifying Mini-Tn5-*bla* to make chromosomal full length SteA-Bla fusion |
| Full length SteA-Bla reverse | TCTGATTTCTAACAAAACTGGCTAAACATAAACGCTTTTTA CTGTCTCTTATACACATCTGGT | For amplifying Mini-Tn5-*bla* to make chromosomal full length SteA-Bla fusion |
| SteA fusion verification primer | GAAAGGCATCTTGTATGTGCT | Verifying the full length SteA-Bla fusion |
| Full length SteC-Bla forward | GGACTCTTGTGGCTAAGGTATTAAAGGATGAATTAAAAAAA CTGTCTCTTATACACATCTCA | For amplifying Mini-Tn5-*bla* to make chromosomal full length SteC-Bla fusion |
| Full length SteC-Bla reverse | TGCCCCCGGCGATTCGCAGAAAAGAACGGAACTAAATGCTA CTGTCTCTTATACACATCTGGT | For amplifying Mini-Tn5-*bla* to make chromosomal full length SteC-Bla fusion |
| SteC fusion verification primer | TGAATATGGCCAATGACGACA | Verifying the full length SteC-Bla fusion |
| fpTOMATO forward (KpnI + ribosomal binding site) | GGTACCTAAGGAGGATATTCATATGGTGAGCAAGGGCGAGGAGGTCATCA | For cloning Tomato fluorescent protein with an added Shine-Dalgarno sequence into pWKS30 |
| fpTOMATO reverse (NotI) | GCGGCCGCTTACTTGTACAGCTCGTCCATGCCGTACAGGAACA | For cloning Tomato fluorescent protein with an added Shine-Dalgarno sequence into pWKS30 |
