# Supplementary material for: Analysis of Cells Targeted by Salmonella Type III Secretion In Vivo
Source: PLoS Pathog. 2007 Dec 21;3(12):e196. doi: 10.1371/journal.ppat.0030196 (PMC2151088; doi:10.1371/journal.ppat.0030196)
Supplement: Supplementary file 9 [file ppat.0030196.st004.doc]

| Supplemental Table IV. Antibodies used for FACS analysis. | |  |
| --- | --- | --- |
|  |  |  |
| Antibody | Clone | Source |
| Affinity Purified Rat IgG2a, l anti-mouse CD16/32, blocks Fc | 93 | eBioscience (Cat. #14-0161) |
| APC conjugated Armenian hamster IgG1,l1 monoclonal immunoglobulin isotype control | G235-2356 | BD Bioscience (Cat. #553956) |
| APC conjugated Armenian hamster IgG1, l2 anti-mouse CD11c monoclonal antibody | HL3 | BD Bioscience (Cat. #550261) |
| APC conjugated rat IgG2a, k anti-mouse CD4 | RM4-5 | BD Bioscience (Cat. #553051) |
| APC conjugated rat IgG2a, k monoclonal immunoglobulin isotype control | R35-95 | BD Bioscience (Cat. #553932) |
| APC-Cy7 conjugated rat IgG2b, k anti-mouse GR-1 monoclonal antibody | RB6-8C5 | BD Bioscience (Cat. #557661) |
| APC-Cy7 conjugated rat IgG2b, k monoclonal immunoglobulin isotype control | A95-1 | BD Bioscience (Cat. #552773) |
| APC-Cy7 conjugated rat IgG2a, k anti-mouse CD19 monoclonal antibody | 1D3 | BD Bioscience (Cat. #557655) |
| APC-Cy7 conjugated rat IgG2a, k monoclonal immunoglobulin isotype control | R35-95 | BD Bioscience (Cat. #552770) |
| PerCP-Cy5.5 conjugated rat IgG2a, k anti-mouse CD8a | 53-6.7 | BD Bioscience (Cat. #551162) |
| PerCP-Cy5.5 conjugated rat IgG2a, k monoclonal immunogobulin isotype control | R35-95 | BD Bioscience (Cat. #550765) |
| PerCP-Cy5.5 conjugated rat IgG2b, k anti-mouse CD11b monoclonal antibody | M1/70 | BD Bioscience (Cat. #550993) |
| PerCP-Cy5.5 conjugated rat IgG2b, k monoclonal immunogobulin isotype control | A95-1 | BD Bioscience (Cat. #550764) |
| PE-Cy7 conjugated Rat IgG2a, k anti-mouse F4/80 monoclonal antibody | BM8 | eBioscience (Cat. #25-4801) |
| PE-Cy7 conjugated rat IgG2a, k monoclonal immunoglobulin isotype control | Not designated | eBioscience (Cat. #25-4321) |
| PE-Cy7 conjugated Armenian hamster IgG anti-mouse CD3e monoclonal antibody | 145-2C11 | eBioscience (Cat. #25-0031) |
| PE-Cy7 conjugated Armenian hamster IgG monoclonal immunoglobulin isotype control | eBio299Arm | eBioscience (Cat. #25-4888) |
| Biotin anti-mouse CD115 | AFS98 | eBioscience (Cat. #13-1152) |
| PE-Cy7 conjugated streptavidin | Not designated | eBioscience (Cat. #25-4317) |
